# Efficiency of High-Frequency Pressing of Spruce Laminated Timber Bonded with Casein Adhesives

**DOI:** 10.3390/polym13234237

**Published:** 2021-12-03

**Authors:** Andreas Herzog, Tobias Kerschbaumer, Ronald Schwarzenbrunner, Marius-Cătălin Barbu, Alexander Petutschnigg, Eugenia Mariana Tudor

**Affiliations:** 1 Forest Products Technology and Timber Construction Department, Salzburg University of Applied Sciences, Markt 136a, 5431 Kuchl, Austria; aherzog.htb-b2018@fh-salzburg.ac.at (A.H.); tkerschbaumer.htb-b2018@fh-salzburg.ac.at (T.K.); rschwarzenbrunner.lba@fh-salzburg.ac.at (R.S.); alexander.petutschnigg@fh-salzburg.ac.at (A.P.); 2 Faculty of Furniture Design and Wood Engineering, Transilvania University of Brasov, B-dul. Eroilor nr. 29, 500036 Brasov, Romania; cmbarbu@unitbv.ro; 3 Institute of Wood Technology and Renewable Materials, University of Natural Resources and Life Sciences (BOKU), Konrad Lorenz-Straße 24, 3340 Tulln, Austria

**Keywords:** spruce, laminated timber, casein adhesive, high-frequency press

## Abstract

This study identifies the importance of reducing press times by employing high-frequency pressing of spruce-laminated timber bound with sustainable casein adhesives. Spruce lamellas with dimensions of 12 × 10 × 75 cm were bonded into five-layered laminated timber and then separated into single-layer solid wood panels. Three types of casein (acid casein from two sources and rennin) were used. To compare the effectiveness of the casein formulation, two control samples bonded with polyvinyl acetate (PVAc) adhesive were pressed at room temperature (20 °C) and also with high-frequency equipment. The tests included compression shear strength, modulus of rupture, modulus of elasticity and screw withdrawal resistance on the wood panel surface and in the glue line. The average values of casein-bonded samples compression strengths ranged from 1.16 N/mm^2^ and 2.28 N/mm^2^, for modulus of rupture (MOR) were measured 85 N/mm^2^ to 101 N/mm^2^ and for modulus of elasticity (MOE) 12,200 N/mm^2^ to 14,300 N/mm^2^. The screw withdrawal resistance (SWR) on the surface of the wood panels ranged from 91 N/mm to 117 N/mm and in the adhesive line from 91 N/mm to 118 N/mm. Control samples bonded with PVAc adhesive did not perform better for compression shear strength, MOR and MOE, but for SWR in the adhesive line with 114 N/mm. Casein-bonded spruce timber pressed with HF equipment represents a sustainable new product with reduced press times, hazardous emissions and improved workability.

## 1. Introduction

Casein is the protein of skimmed milk [1], which is obtained by precipitation [2] and represents an alternative adhesive for wood-based composites previously used for bonding such as urea-formaldehyde (UF), phenol-formaldehyde (PF), isocyanates (pMDI), etc. [3]. Synthetic adhesives have better properties, for example, dimensional stability, but contain formaldehyde, even though there are ways to reduce using additives like UF [4,5,6].

The use of casein for wood bonding dates back to ancient Egypt or the Middle Ages [7]. At that time, craftsmen worked with it to build thicker panels from thin parts for paintings [8], for producing violins [9]. The plywood used for aircraft in the First World War was bonded with casein adhesive [10,11] due to its appropriate bond strength under exterior use conditions. This natural protein polymer was used as an adhesive for laminated timber for centuries [12]. The glue-laminated timber-glulam (GLT) portal frame bonded with casein adhesive was a patent registered by carpenter Otto Hetzer in 1906 [13], as casein was included in the category of structural wood adhesives [14]. Later on, waterproof synthetic phenolic formaldehyde resins were introduced and casein adhesive was used to a lesser extent. In current applications, casein adhesives are employed for gluing bottle labels or cigarette papers [7,15], high-quality paper finishing antistatic varnishes of natural and synthetic fibers, indoor applications [16]. The basic requirements of a casein adhesive, procurable as a powder [3], are water and mold resistance, mingled with suitable dry strengths. When supplementary properties should be acquired, the casein adhesive formulations should be prepared considering the natural origin, cheapness, low-staining tendencies, long service life, high dry strength, or good spreading characteristics, even with some diminution of water resistance [17]. To solubilize the casein, it should be soaked in water; after it has swollen, it is blended with alkali as lime [7]. The pot life of casein adhesive can be extended by adding sodium silicate [16]. Urea and ammonia influence a decrease in viscosity by reducing the hydrogen bonds [18]. There are some examples of research conducted with solid wood bound with casein. Konnerth et al. (2006) [19] compared dry bond strength of spruce and beech wood bonded with diverse adhesives, among which was casein (no information about the casein formulation was given). By applying the testing method of scarf- and lap joint, no clear effect of the different adhesives was observed for lap joint. The scarf-joint testing resulted in the shear strength of the samples bonded with casein being lower than that bound with polyvinyl acetate (PVAc), melamine urea-formaldehyde (MUF), phenol–resorcinol–formaldehyde resin (PRF) and one-component polyurethane adhesive (PUR). Mitani and Barboutis (2016) [20] investigated the bonding strength of PVAc and casein adhesives in small diameter beech wood. The components of the casein adhesive were casein milk, lime, potash, chalk, borates, carbonates, silicates and caseinates. The blending ratio of the adhesive powder and water was 1:1, but no information about the percentage share was given. It was found that the modulus of rupture (MOR) was influenced by the type of adhesive and the use of PVAc returned better values of MOR compared to bonding with casein. The formulation of casein adhesive significantly influences the mechanical and physical properties of the end-product [3]. One of the disadvantages of casein formulations is the long pressing time needed at room or lower temperatures [3].

The aim of this work was to analyze the bonding quality with casein adhesive of laminated timber by employing a high-frequency press and comparing this process with cold pressing with a hydraulic veneer press. The press time with high-frequency (HF) press is reduced considerably, the radio-frequency shortens the curing process of the adhesive from hours to minutes [12]; this is a process that has been used for gluing wood for a long time [21]. The HF heating and pressing system has already commercially been used in the manufacturing of glued laminated board or laminated veneer lumber (LVL) [22,23]. During this bonding, the wood lamellae are exposed to a high-frequency field [24] and the water molecules in this area are accelerated to oscillate, which creates heat at the location of the molecules. An important requirement is that the surface to be bonded should have a higher moisture content (m.c.) than the neighboring media. The effect of high humidity is rapid heating and evaporation of the water and subsequently faster adhesive hardening [24].

## 2. Materials and Methods

Milled spruce lamellae (*Picea abies*) with 120 mm × 1500 mm × 20 mm and an m.c. of 12% were provided by the company Weinberger-Holz (Reichenfels, Austria). The spruce lamellas were bonded to 5-layer laminated timber with PVAc type D3, casein-based adhesives (with acid casein and rennin) and then cut into test specimens. Before pressing, the spruce lamellas were stored at 18 °C and 50% relative air humidity for at least seven days.

**Figure 1:**
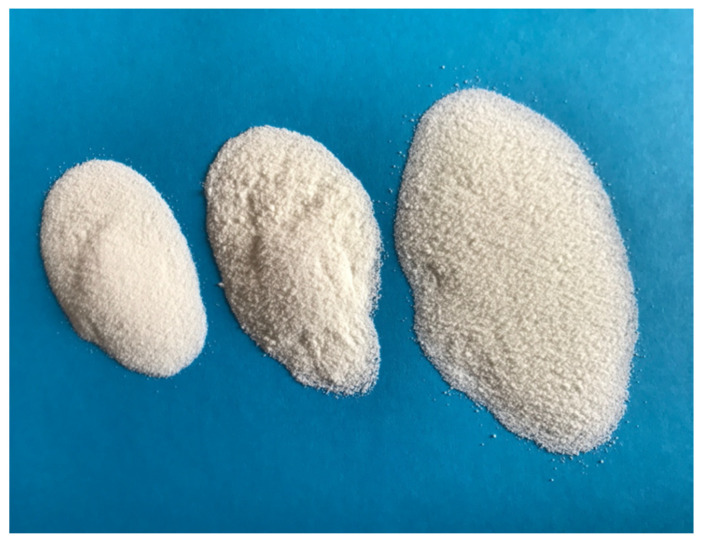
Three types of casein used in this study (left = acid-casein of Kremer Pigmente Co., middle = acid-casein of Woerle Co., right = rennin of Woerle Co.

The casein adhesive formulation builds on [3]. Two lactic acid precipitated casein types from the companies Kremer Pigmente (Aichstetten, Germany), dairy Gebrüder Woerle (Salzburg, Austria) and rennin of the same company (Gebrüder Woerle, Salzburg, Austria) were used (Figure 1).

The formulation for casein adhesive also includes water (pH value 7) and marble pit lime (deposited for three months and composed of white hydrated lime, solids content 40%) from Baumit Co. (Wopfing, Austria). Subsequently, the lime was added until a homogeneous mass was formed. The ingredients were manually mixed at room temperature of 20 °C and 35% relative air humidity in a hard-plastic recipient and stirred with a mechanical mixer at a speed between 700 and 1500 rpm. A typical wood adhesive (PVAc type D3) from Würth Co. (Böheimkirchen, Germany) was used as reference in both pressing processes (cold and HF).

The samples were labeled as follows: the first letters describe the production process, e.g., “HF” for high frequency pressing with casein and “CP” for cold pressing (20 °C) with OTT veneer press (Maschinen-Grup, Nattheim, Germany). The next letters describe the type of casein used, such as “AK” for acid casein from Kremer Pigmente Co. or “RW” for rennin Woerle Co. In addition, the samples were numbered with the consecutive production number. These numbers are also connected with casein formulation, such as more water content or specifications concerning adhesive application. All casein formulations, press type, press time and adhesive application presented in this study are summarized in Table 1. The formulation CP-AK1 [3] serves as the basis, due to the highest values for the quality of the bonding reported in previous studies, compared to other casein adhesive formulations. For a better adhesive application, a formulation containing 5% more water was used (CP-AK2), with a consistency similar to PVAc adhesive.

**Figure 2:**
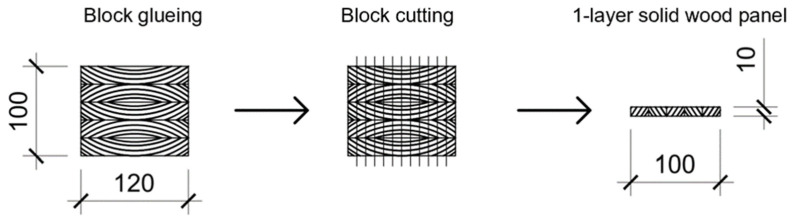
Schematic representation of a single-layer solid wood panel production in a block process from laminated timber.

The spruce lamellas were first bonded into 5-layer laminated timber (100 × 120 × 750 mm) (height × width × length). Eight solid wood panels were manufactured. In order to generate a higher number of test specimens, the laminated timber was separated into single-layer solid wood panels (Figure 2). From a single-layer solid wood panel, 9 samples were cut.

**Figure 3:**
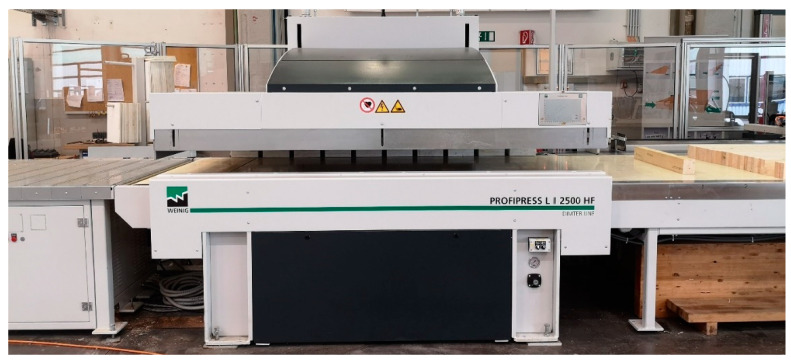
High-frequency press used by Weinig Dimter Co. (Profipress L2 2500 HF, type PPL2-2500) as trial equipment at its research center in Illertissen, Germany.

The production of the spruce glue-laminated samples (series HF) with the high-frequency press (Profipress L2 2500 HF, machine type PPL2-2500 of Weinig Co.; Illertissen, Germany) which has a 30-kW high-frequency generator (Figure 3) was carried out at Weinig Dimter Co. in Illertissen (Germany).

To achieve faster pressing times with casein adhesive, seven samples were bonded in an HF-press. In addition, one sample (HF-PVAc-D3) was produced as a reference with PVAc-D3 (Kleiberit, Weingarten, Germany), with pH level 3 and viscosity 12,000 ± 2000 mPa × s).

The samples were pressed in HF with 1 N/mm^2^. During the tests, 2 or 4 min of pressing time were set (Table 1). The ambient conditions were 19.6 °C and 34% relative air humidity. All samples were produced individually with the same parameters of the HF press equipment: power 6 kW, amperage 2 to 3 A, electrical voltage 4000 V and frequency 3 MHz.

**Figure 4:**
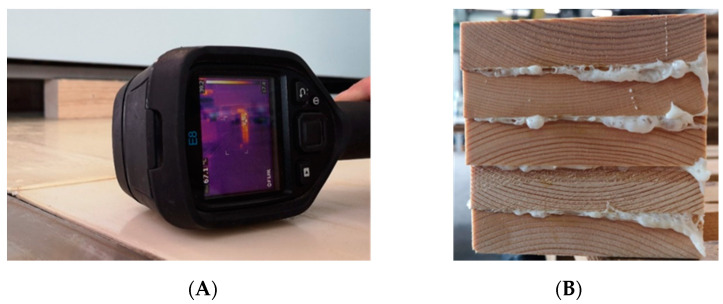
Temperature measurement in the adhesive line during high-frequency pressing by means of a thermal imaging camera (Flir E8) (**A**). Casein glued bonded 5-layer laminated timber block, just after high-frequency pressing (**B**).

During the pressing process, the heat development in the adhesive line was monitored with a thermal imaging camera Teledyne Flir E8 (Wilsonville, OR, USA) (Figure 4A). The temperature curve was recorded at intervals of 15 s. The casein adhesive reached a temperature of 60 °C.

**Figure 5:**
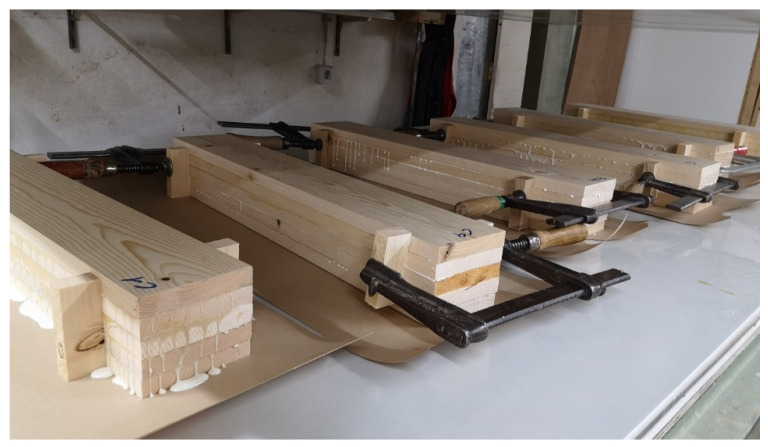
Production of the spruce laminated timber blocks (5 × 20 × 120 × 750 mm) in the cold process before pressing.

As a comparison to the HF technology, a series of samples was produced with a hydraulic veneer cold press (CP). The samples were manufactured using an OTT veneer press at the joinery Möbel Scheiber Co. (Leogang, Austria). The samples were placed consecutively in the press until a pressure of 30 bar was reached. The glued solid wood blocks were pressed for 24 h at a room temperature of 22 °C and 40% relative air humidity. The spruce blocks were additionally secured against lateral slipping with screw clamps (Figure 5).

Physical and mechanical properties of the test samples bonded with casein adhesives and PVAc for references were according to European norms (EN). Before testing, all test samples were conditioned at room temperature (21 °C and 52% relative air humidity). The m.c. of the samples was 11%. The tests for mechanical properties were carried out on Salzburg University of Applied Sciences (SUAS) at Campus Kuchl using the testing machine Zwick Roell Z250 (Ulm, Germany).

To determine the compression shear strength according to EN 13354:2009 [25] the water-resistance of the single-layer solid wood panels (Figure 2), ten specimens were tested before and ten of them after 24 h of water storage at room temperature. The test specimens were cut according to [25] with a shear width of 10 ± 5 mm, a shear length of 25 ± 5 mm and a saw blade cut width of 3 mm. The compression shear strength was determined under a constant loading rate set at 2 mm/min and the maximum load at failure was recorded.

Ten specimens from each single-layer solid wood panel (Figure 2) were cut with 250, 50 and 10 mm length, width and thickness, respectively, to determine the 3-point bending strength (MOR) and modulus of elasticity (MOE) according to EN 310:2005 [26]. Test specimens were all tested in dry state.

The determination of the screw-withdrawal resistance according to EN 320:2011 [27] was carried out for 20 specimens with dimensions 50 mm width and 10 mm thickness. Each adhesive composition was tested both in the wooden core and in the adhesive line of the single-layer solid wood panel (Figure 2).

When selecting the test specimens, the cutting plan was chosen in such a way that the specimens were evenly distributed through the board, according to EN 326-1:2005.

The results were statistically analyzed using IBM SPSS software with one-way ANOVA and post Tukey HSD.

## 3. Results and Discussion

The tests carried out to determine the properties of laminated timber bound with three types of casein, using several formulations, compared with control samples bonded with PVAc, are: compressive shear strength, bending strength (modulus of rupture) and modulus of elasticity in bending and screw withdrawal resistance.

### 3.1. Compression Shear Strength

**Figure 6:**
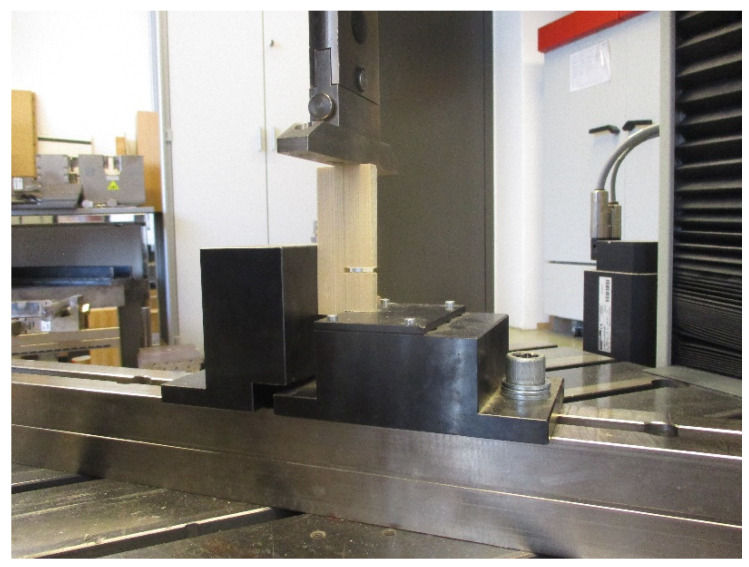
Shear strength test of the single-layer solid spruce laminated timber.

The most important test for adhesives is the compressive shear strength after 24 h of water storage (Figure 6). In this test, the sample CP-AK2 showed the highest compressive shear strength (Table 2). This testing specimen has 5% more water in the casein adhesive formulation than the reference CP-AK1. This is also a significant advantage for the processability of the adhesive due to its consistency. In addition, these samples achieved 50% higher shear strengths than the reference sample CP-PVAc-D3.

The more interesting results of this study are the samples produced in an HF press. The coefficient of variation for all the tested samples ranges from 0.08% to 0.31%.

During the high-frequency bonding, the workpiece was exposed to a high-frequency stress field. The water molecules in this field were turned on to oscillate, similar to the principle of a microwave. The 12% moisture content (suitable for this process) of the lamellae leads to faster heating and evaporation of the water and, as a result, faster hardening of the adhesive.

The highest value was achieved by the sample HF-AK5, with a maximum of 1.94 N/mm^2^. Its HF pressing time was 4 min. In addition, the adhesive was utilized with an adhesive application machine and half the amount of resin (200 g/m^2^) (Table 1). For HF-AK5, the shear strength is only 10% lower than CP-AK2 (adhesive application 200 g/m^2^ and pressing time 24 h). In addition, the testing specimen HF-AK5 achieved 15% higher compressive shear strength values than HF-PVAc-D3. It is also striking to note that HF-AK5 measured 9% higher values than HF-AK4, which was produced by using a resin filler and with a higher adhesive application (400 g/m^2^). It is interesting how an increase in the water amount by 5% has a positive effect on the compressive shear strength after 24 h of water storage. This is clearly recognizable in the comparison of HF-AK2 and HF-AK4, both pressed with the same pressing parameters. The shear strength was negatively affected by pressing for only 2 min using the high-frequency method. Given the high-water content, the 2-min pressing time did not allow a complete heating and evaporation of the water and consequently appropriate curing of casein adhesive.

The results of the sample CP-AK5 should not be disregarded. This sample also contains 5% more water in the formulation. The adhesive was applied after 90 min. The results showed that this procedure had no effect on the pressing time.

The use of the adhesive application machine with resin spreader with roller applicator is also interesting. By using this method, the adhesive could be spread more homogeneously than with manual application. This also affects the results positively. In addition, the resin amount could be reduced by half.

When comparing the different types of casein, the processing of rennin in the adhesive formulation turned out to be difficult. The breakdown of the casein-water mixture by the lime occurs abruptly after its addition. Despite constant stirring, lump formation in the mixture cannot be avoided and thus an even adhesive application on the material cannot be achieved. The high scatter in the values of the samples produced with rennin (HF-RW and CP-RW8) can be attributed to these application-related aspects.

Although these samples achieved high values in the compression shear test after 24 h of water storage. It can be concluded that the uneven adhesive distribution in both the high-frequency press and the cold-pressing process has no effect on the water-resistance of the adhesive line.

Using acid casein from Woerle Co., the sample CP-AW7 was produced in the cold pressing process. In the compression shear test after water storage, this type of casein achieved the second-highest values using the cold press method, while this sample showed the lowest values before water storage. Increasing the water amount by 5% proves that satisfactory results can be obtained in both areas when the appropriate formulation is used.

### 3.2. Modulus of Rupture and Modulus of Elasticity in Three-Point Bending Test

The modulus of rupture (MOR) of all test specimens differs only slightly (Table 3), with a reduced coefficient of variance of 0.03% to 0.09%. The press procedures and recipe-related changes have no significant influence on the flexural strength (Table 3). The highest value was achieved by the samples pressed with HF (HF-RW and CP-RW) and with rennin in the casein formulation (110 N/mm^2^). In this case, there is a narrow difference of 4% between MOR in the case of CP (101 N/mm^2^) compared to HF (97 N/mm^2^) for the mean values of the specimens. The lowest value was obtained with HF-AK5. The specimens CP-AK1 and HF-AK4 have a lower bulk density, which explains the lower values of MOR.

The results of modulus of elasticity (MOE) in the three-point bending test differ to some extent. The highest value was achieved by the sample CP-AK3. The casein adhesive was heated to 30 °C before pressing in order to simulate higher temperatures during summer in industrial production halls. The lowest value of the modulus of elasticity in bending is HF-AK5. This result could be due to the adhesive and resin application of 200 g/m^2^ (Table 1), half of the amount referred for the rest of the spruce laminated timber samples. The increase in water content, as seen in the samples CP-AK1 and CP-AK2, has no significant effect on the elasticity. This is also reflected in the MOR of the samples HF-AK2 and HF AK-4, which were pressed four minutes in the high-frequency press.

Regarding the MOE in the three-point bending test, the variation coefficient is between 0.02% and 0.18%. Similar to the compression shear test, the specimens with rennin achieved high values both in the cold pressing process (mean value of 14,238 N/mm^2^) and in the HF press (mean value of 13,830 N/mm^2^). Only the sample CP-RW6 showed significantly lower values, from which it can be concluded that the use of rennin in the cold pressing process requires a higher water amount in the formulation. In the high-frequency process, on the other hand, high values were obtained despite a lower water content.

### 3.3. Screw Withdrawal Resistance

No significant influence of the adhesive joint can be determined for the screw withdrawal resistance (SWR) (Table 4). The coefficient of variation for SWR in the wooden core was from 0.005% to 0.21% and for the adhesive line from 0.03% to 0.15%. The specimens bound with casein adhesive have on average higher SWR values in wood and in the adhesive line. When SWR was tested in the wood core, the maximum results were measured for the sample HF-AK5 (145 N/mm), followed by the specimen with rennin in the formulation HF-RW and CP-AK4, both with 141 N/mm. The higher mean values were measured for the specimens pressed with screw clamps (117 N/mm) for CP-AK1, followed by the series pressed with HF: 115 N/mm for HF-AK1 and 114 N/mm for HF-AK5. The two PVAc test groups also have higher maximum values when tested in the adhesive line, but are comparable with SWR of the HF samples pressed for 2 (HF-AK1) and 4 min (HF-Ak2), respectively. The highest mean of SWR (118 N/mm) was calculated for the sample HF- AK1, followed by 114 N/mm for the reference PVAc-D3. For the samples pressed with screw clamps, CP-AK1 and CP-AK3 SWR in the adhesive line was 112 N/mm. A small decrease in SWR was recorded for the sample with PVAc (109 N/mm) when pressed with HF, followed by the specimens pressed with HF and with acid casein in the adhesive formulation HF-AK2 and HF-AK5 (108 N/mm). There are no significant differences between SWR tested in the wood surface and in the adhesive line. The mean values measured in the wooden core ranged from 90 to 117 N/mm, while in the adhesive line a similar interval of 91–118 N/mm was recorded. The mean SWR of control samples (PVAc-D3 and HFPVAc-D3) did not overcome the values of the samples

Since the annual ring length of the lamellae were not considered in the production of the solid wood blocks, certain results of this test series can be influenced.

## 4. Conclusions

In this research study, we analyzed the influence of the pressing method (cold pressing and high-frequency press with industrial equipment) on spruce laminated timber bound with casein adhesive (with different formulations) and PVAc type D3, as control samples.

The consequence of volumetric heating with a high-frequency press was a faster moisture leveling and a more uniform resin cure, which lead to an agile and more effective hot-pressing process. Moreover, the pressing time was reduced significantly, from 24 h to 2 and 4 min, which represents a decrease in 99% of the press time, employing energy and improvement of production time.

With an increase in the water amount of 5%, due to reduced viscosity, the processability improves without affecting the mechanical properties. In this way, the casein adhesive has a similar consistency as PVAc, which represents an important criterium for good spreading with specialized equipment. Moreover, the pot life of the casein adhesive should be extended to more than two hours. It was also proven that the casein formulation should be strictly monitored because the mechanical properties can worsen with an increased water amount.

By combining the results, the industrial processing of lamellae bound with casein adhesive can be prefigured. The mechanized adhesive application improves the bonding quality through a uniform distribution. By employing high frequency, the press time is considerably reduced to less than five minutes. There is therefore high potential for optimization of press time and the adhesive amount.

A certain degree of moisture content at the board surfaces would be interesting to include in further studies, for the improvement of heat transfer to the middle layer during pressing.

The next research will compare the water resistance and the thermo-resistance of the adhesive lines from both the casein and PVAc adhesives and the influence of the width of lamellae annual rings and their thickness on the mechanical and physical properties. Additionally, the influence of the moisture content of the surface to be bonded will be analyzed in a forthcoming study. Research is also in progress regarding the improvement of the casein formulation to extend the pot life of the adhesive because the application time is limited due to a quick increase in viscosity after the reaction and the settle time of all the ingredients.

Casein-bound spruce laminated timber pressed with high-frequency equipment represents a sustainable and recyclable new product, easily integrable in green construction solutions.

## Figures and Tables

**Table 1:** Adhesive formulations in parts by weight (pbw) and pressing parameters for the bonding of 5-layer laminated timber.

Sample	Press Process	Press Time	Casein Type	Casein (pbw)	Water (pbw)	Lime (pbw)	Adhesive Amount (g/m^2^)
PVAc-D3	CP	24 h	-	-	-	-	200
HFPVAc-D3	HF	4 min	-	-	-	-	200
HF-AK1	HF	2 min	Acid (Kremer)	1.5	3.5	1.0	400
HF-AK2	HF	4 min	Acid (Kremer)	1.5	3.5	1.0	400
HF-AK3	HF	2 min	Acid (Kremer)	1.5	4.5	1.0	400
HF-AK4	HF	4 min	Acid (Kremer)	1.5	4.5	1.0	400
HF-AK5	HF	4 min	Acid (Kremer)	1.5	4.5	1.0	200
HF-RW	HF	4 min	Rennin (Woerle)	1.5	3.5	1.0	400
HF-AW	HF	4 min	Acid (Woerle)	1.5	3.5	1.0	400
CP-AK1	CP	24 h	Acid (Kremer)	1.5	3.5	1.0	400
CP-AK2	CP	24 h	Acid (Kremer)	1.5	4.5	1.0	400
CP-AK3 *	CP	24 h	Acid (Kremer)	1.5	3.5	1.0	400
CP-AK4 **	CP	24 h	Acid (Kremer)	1.5	4.5	1.0	400
CP-AK5 ***	CP	24 h	Acid (Kremer)	1.5	3.5	1.0	400
CP-RW6	CP	24 h	Rennin (Woerle)	1.5	3.5	1.0	400
CP-AW7	CP	24 h	Acid (Woerle)	1.5	3.5	1.0	400
CP-RW8	CP	24 h	Rennin (Woerle)	1.5	6.5	1.0	400
CP-AW9	CP	24 h	Acid (Woerle)	1.0	2.5	1.0	400

* Heating of the casein adhesive before blending at approx. 30 °C. ** Adhesive application after 90 min and increase in the water content. *** Adhesive application after 45 min and use of casein reference adhesive.

**Table 2:** Shear strength of single-layer solid spruce laminated timber (values with the same letter (a, b, c, d) are not significantly different ANOVA, post Tukey HSD, *p* = 0.05, standard deviation in parentheses).

Shear Strength (N/mm^2^)				
Sample	Mean	Minimum	Maximum	*p*
PVAc-D3	1.17 ^a^ (0.24)	0.84	1.60	
HFPVAc-D3	1.77 ^c^ (0.30)	1.38	2.21	.
HF-AK1	1.41 ^b^ (0.28)	0.97	1.87	.
HF-AK2	1.53 ^b^ (0.46)	0.70	2.19	.
HF-AK3	1.16 ^a^ 0.36)	0.65	1.69	
HF-AK4	1.88 ^c^ (0.21)	1.60	2.28	.
HF-AK5	2.07 ^d^ (0.40)	1.40	2.81	.
HF-RW	1.66 ^c^ (0.27)	1.23	2.07	.
HF-AW	1.32 ^b^ (0.29)	1.03	1.81	.
CP-AK1	2.18 ^d^ (0.18)	1.93	2.41	.
CP-AK2	2.28 ^d^ (0.39)	1.25	2.65	.
CP-AK3	1.74 ^c^ (0.21)	1.47	2.12	.
CP-AK4	2.10 ^d^ (0.16)	1.91	2.33	.
CP-AK5	1.69 ^c^ (0.15)	1.45	1.94	.
CP-RW6	1.52 ^b^ (0.22)	1.11	1.87	.
CP-AW7	1.54 ^b^ (0.25)	1.23	2.06	.
CP-RW8	1.45 ^b^ (0.22)	0.92	1.81	.
CP-AW9	1.47 ^b^ (0.27)	1.17	2.08	.

Values are significantly different from the control (ANOVA, *p* < 0.05).

**Table 3:** Modulus of rupture and modulus of elasticity in bending of single-layer solid spruce laminated timber (values with the same letter (a, b, c, d) are not significantly different ANOVA, post Tukey HSD, *p* = 0.05, standard deviation in parentheses).

Modulus of Rupture (N/mm^2^)					Modulus of Elasticity (N/mm^2^)			
Sample	Mean	Min.	Max.	*p*	Mean	Min.	Max.	*p*
PVAc-D3	98 ^d^ (7.7)	88	106	.	13,706 ^c^ (1441)	11,641	15,301	.
HFPVAc-D3	89 ^a^ (8.3)	80	104		12,177 ^b^ (2214)	10,363	15,302	
HF-AK1	93 ^b^ (3.3)	87	97	.	13,265 ^c^ (369)	12,279	13,607	.
HF-AK2	91 ^b^ (2.8)	83	93	.	13,244 ^c^ (336)	12,543	13,732	.
HF-AK3	90 ^b^ (4.9)	84	100	.	13,187 ^c^ (790)	12,283	14,149	.
HF-AK4	86 ^a^ (2.6)	81	90		12,484 ^b^ (423)	11,842	13,205	
HF-AK5	85 ^a^ (4.2)	77	91		10,614 ^a^ (1054)	9260	12,260	
HF-RW	97 ^d^ (9.0)	83	110	.	13,830 ^c^ (1025)	12,521	15,121	.
HF-AW	94 ^b^ (3.9)	89	101	.	12,685 ^b^ (661)	11,185	13,352	.
CP-AK1	90 ^b^ (3.2)	86	96	.	12,237 ^b^ (340)	11,749	12,739	.
CP-AK2	89 ^a^ (5.6)	81	95		12,298 ^b^ (1180)	10,665	13,411	
CP-AK3	97 ^d^ (4.4)	92	105	.	14,342 ^d^ (560)	13,635	15,293	.
CP-AK4	92 ^b^ (3.8)	88	101	.	12,961 ^b^ (520)	11,964	13,681	.
CP-AK5	93 ^b^ (3.5)	85	97		13,866 ^c^ (392)	13,019	14,341	.
CP-RW6	90 ^b^ (7.6)	78	104		12,131 ^b^ (1202)	9225	15,469	.
CP-AW7	95 ^c^ (6.2)	84	106		13,753 ^c^ (901)	11,824	14,905	.
CP-RW8	101 ^d^ (7.7)	89	110		14,238 ^d^ (1143)	12,338	15,395	.
CP-AW9	96 ^d^ (5.0)	88	102		14,137 ^d^ (482)	13,323	15,043	

Values are significantly different from the control (ANOVA, *p* < 0.05).

**Table 4:** Screw withdrawal resistance (in wood and in the adhesive line) of single-layer solid spruce laminated timber (values with the same letter (a, b, c, d) are not significantly different ANOVA, post Tukey HSD, *p* = 0.05, standard deviation in parentheses).

	Screw Withdrawal Resistance (N/mm)							
	Wood				Adhesive Line			
Sample	Mean	Min.	Max.	*p*	Mean	Min.	Max.	*p*
PVAc-D3	98 ^b^ (8.7)	9127	117	.	114 ^d^ (13.4)	92	132	.
HFPVAc-D3	102 ^b^ (5)	93	108	.	109 ^c^ (9.5)	99	125	.
HF-AK1	115 ^d^ (10.5)	97	130	.	118 ^d^ (9)	104	128	.
HF-AK2	99 ^b^ (14)	90	133	.	108 ^c^ (17)	83	131	.
HF-AK3	92 ^a^ (5.3)	86	101		91 ^a^ (5)	85	99	
HF-AK4	95 ^a^ (3.8)	87	100	.	103 ^b^ (9.4)	89	123	.
HF-AK5	114 ^d^ (23)	81	145	.	108 ^c^ (7)	98	122	.
HF-RW	108 ^c^ (23.4)	78	141	.	106 ^c^ (12)	86	121	.
HF-AW	101 ^b^ (8.1)	85	108		103 ^b^ (10)	90	117	.
CP-AK1	117 ^d^ (13.1)	103	141	.	112 ^d^ (5.3)	104	120	.
CP-AK2	104 ^b^ (2.7)	98	108		104 ^b^ (6)	98	114	.
CP-AK3	97 ^b^ (10.5)	85	110		112 ^d^ (6)	100	121	.
CP-AK4	110 ^c^ (16.3)	93	141	.	103 ^b^ (8.6)	96	125	.
CP-AK5	91 ^a^ (5.5)	80	100	.	99 ^b^ (3)	96	106	.
CP-RW6	94 ^a^ (6.4)	85	107	.	103 ^b^ (15)	84	129	.
CP-AW7	95 ^a^ (7.3)	83	107	.	96 ^a^ (6)	87	104	
CP-RW8	90 ^a^ (9)	75	102	.	95 ^a^ (8.3)	84	108	
CP-AW9	108 ^c^ (8.3)	98	120	.	96 ^a^ (11.2)	82	116	.

Values are significantly different from the control (ANOVA, *p* < 0.05).
